# Recent advances in nuclear medicine and their role in inflammatory arthritis: focus on the emerging role of FAPI PET/CT

**DOI:** 10.1007/s00256-024-04834-w

**Published:** 2024-11-25

**Authors:** Christian Schmidkonz, Torsten Kuwert, Theresa Ida Götz, Andreas Ramming, Armin Atzinger

**Affiliations:** 1 Department of Nuclear Medicine, Erlangen University Hospital, Erlangen, Germany; 2 Institute for Medical Engineering, Ostbayerische Technische Hochschule Amberg-Weiden, Amberg, Germany; 3 Department of Internal Medicine 3, Rheumatology and Immunology, Erlangen University Hospital, Erlangen, Germany; 4 Deutsches Zentrum Fuer Immuntherapie (DZI), Erlangen University Hospital, Erlangen, Germany

**Keywords:** FAPI, PET/CT, Molecular imaging, Joint diseases

## Abstract

Imaging molecular processes associated with inflammatory disease has been revolutionized by hybrid imaging using positron emission tomography/computed tomography (PET/CT). PET/CT visualizes metabolic activity as well as protein expression and provides a comprehensive whole-body evaluation. It has the potential to reveal inflammation prior to detection of structural changes in inflammatory joint diseases. FAP is a type II transmembrane glycoprotein overexpressed not only in the stroma of tumors but also in the fibrotic processes of certain immune-mediated disorders. The recent introduction of fibroblast activation protein inhibitors (FAPI) labeled by positron emitters and thus suitable for PET/CT allows to investigate FAP expression in vivo. This review will focus on the use of FAPI-PET/CT for the diagnosis and evaluation of treatment response in inflammatory joint diseases.

## Introduction

Imaging plays an important role in the diagnosis and evaluation of treatment response in inflammatory bone diseases such as rheumatoid arthritis (RA), psoriasis arthritis (PsA), or ankylosing spondylitis. X-ray imaging is frequently employed to identify joint damage but is insensitive to early inflammatory changes [1]. Magnetic resonance imaging (MRI) with gadolinium-labeled contrast media is valuable to detect inflammation, although resource-demanding and presenting constraints in terms of whole-body evaluation [2]. In superficial structures, ultrasound allows real-time assessment of joint inflammation, though its efficacy may hinge on the operator’s skills [3]. X-ray computerized tomography (CT) visualizes bone anatomy at high resolution and is, thus, eminently suitable to detect bone destruction. However, all these methods do not visualize the process of mesenchymal stromal activation in inflammatory joint diseases and, therefore, do not allow the detection of the molecular process underlying and preceding tissue destruction itself.

Imaging molecular processes associated with inflammation in vivo has been revolutionized by hybrid imaging using PET/CT providing complementary molecular and structural information. PET/CT visualizes and quantifies metabolic activity as well as protein expression in vivo and provides a comprehensive, whole-body evaluation, potentially revealing inflammation prior to structural changes in inflammatory joint diseases.

To date, the most common tracer used for this application is ^18^F-fluorodeoxyglucose (^18^F-FDG) which allows the detection of inflammatory lesions based on their increase in glycolytic metabolism [3–5]. Numerous studies regarding the use of ^18^F-FDG PET/CT in inflammatory joint diseases such as RA have been published. These are beyond the scope of this review which focuses on FAPI PET/CT. The following, therefore, gives only a brief summary of the use of ^18^F-FDG PET/CT in inflammatory joint disease.

### ^*18*^*F-FDG PET/CT in rheumatoid arthritis*

In a study by Bhattarai et al., a visual scoring system and quantitative parameters such as maximum standardized uptake value (SUVmax), metabolic active volume (MAV), and total lesion glycolysis (TLG) were used to differentiate RA from other arthropathies [6].

Lee et al. explored the correlation of ^18^F-FDG PET/CT-derived parameters in 68 joints with disease activity and compared the reliability of joint counts between ^18^F-FDG PET/CT and clinical assessments in patients with RA [7]. The authors enrolled a total of 91 patients and found that the number of PET-positive joints was significantly correlated with the swollen joint count, the tender joint count, and the disease activity score in 28 joints. Intra- and interobserver reliability of PET for the affected joint counts was excellent.

Ravikanth et al. demonstrated that ^18^F-FDG PET/CT is also a valuable tool for the evaluation of treatment response in patients suffering from RA undergoing therapy [8]. These authors studied 42 patients undergoing treatment with anti-tumor necrosis factor α that were assessed using whole-body ^18^F-FDG PET/CT before and 3–6 months after therapy. They found a significant correlation between ΔSUV and disease activity scores after 6 months of treatment.

In a randomized controlled trial, Bouman et al. recruited 79 patients with low disease activity of RA that were assessed with ^18^F-FDG PET/CT to apply PET parameters to predict the outcome of tapering TNF-inhibitor (TNFi) treatment for 18 months [9]. This study suggested that ^18^F-FDG PET/CT could detect clinical disease activity in patients with clinically low disease activity or remission. However, no predictive values were found for FDG-PET parameters for successful tapering and/or discontinuation of TNFi.

The assessment of both the synovial and aortic inflammation in patients suffering from RA is possible by ^18^F-FDG PET/CT as demonstrated by Hamar et al. [10]. It was shown that patients taking tofacitinib had suppressed disease activity as determined by PET/CT as well as a reduction in serologic parameters and clinical disease activity scores.

That ^18^F-FDG PET/CT has a potential role in patients with early rheumatoid arthritis following the initiation of combination therapy with triple oral antirheumatic drugs was demonstrated by Roivainen et al. [11]. The study involved 17 patients with active RA in whom combination therapy was initiated with methotrexate, sulfasalazine, hydroxychloroquine, and low-dose oral prednisolone. Clinical disease activity was assessed at screening, at baseline, and after 2, 4, 8, and 12 weeks of therapy. ^18^F-FDG PET/CT of all joints was performed at baseline and after 2 and 4 weeks of therapy. ^18^F-FDG maximum standardized uptake values showed a significant reduction during therapy and correlated with treatment efficacy as well as clinical outcome in patients with early RA. The authors concluded that ^18^F-FDG PET/CT may help predict the therapeutic response to novel drug treatments; however, due to the small number of patients enrolled, larger prospective trials are warranted.

### FAPI, a novel promising radiotracer

Although the detection of active inflammation by ^18^F-FDG PET/CT is implemented in clinical routine, the detection of tissue response and tissue remodeling processes, which accompany immune-mediated inflammatory diseases and lead to organ damage, has until recently not been possible [12, 13]. In order to evaluate metabolic pathways other than glucose metabolism, several PET tracers have been proposed as an alternative to ^18^F-FDG. The most promising radiotracers that have emerged in the last few years are fibroblast activation protein inhibitors labeled with either ^68^Ga or ^18^F [14–16]. Fibroblast activation protein-α is a type II transmembrane protein that is overexpressed in activated fibroblasts. These cells play a crucial role in a wide range of pathophysiological conditions such as the development of the tumor microenvironment, which is involved in tumor growth, migration, progression, wound-healing, and inflammation [17–21]. The role of FAPI PET/CT for the evaluation of cancer has been evaluated in a large number of studies with promising results [22–24]. However, fibroblast activation is occurring not only in neoplasms but also in immune-mediated inflammatory lesions. Tissue remodeling during inflammation is based on mesenchymal stroma cell activation and expansion in the synovial membrane of inflamed joints. Moreover, catabolic FAP-positive extracellular matrix-degrading phenotypes of fibroblasts are associated with cartilage destruction and bone erosions, as seen in rheumatoid arthritis [25]. Tissue remodeling is also a consequence of chronic inflammation and the critical step for eliciting damage, eventually causing disability if not diagnosed and treated in time. One of the advantages of the use of FAPI imaging for bone lesions is that bone marrow usually exhibits low physiological FAPI uptake [4, 26]. FAPI PET is independent of glucose activity, leading to the drastic reduction of background signal in the brain, liver, oro- and nasopharyngeal mucosa, or gastrointestinal tract. In practical use, ^68^Ga-FAPI can be used without any dietary preparation and provides stable tracer uptake 10 min to 3 h after administration.

Preliminary evidence has generated raging scientific interest in FAP as the next billion-dollar nuclear theranostics target in the field of nuclear medicine even if the evidence in the literature is still in its early stages [27]. Therefore, in this concise review, we would like to present the current status of FAPI PET/CT for the diagnosis and evaluation of treatment response in inflammatory joint diseases.

### FAPI PET/CT in inflammatory joint diseases

**Fig. 1:**
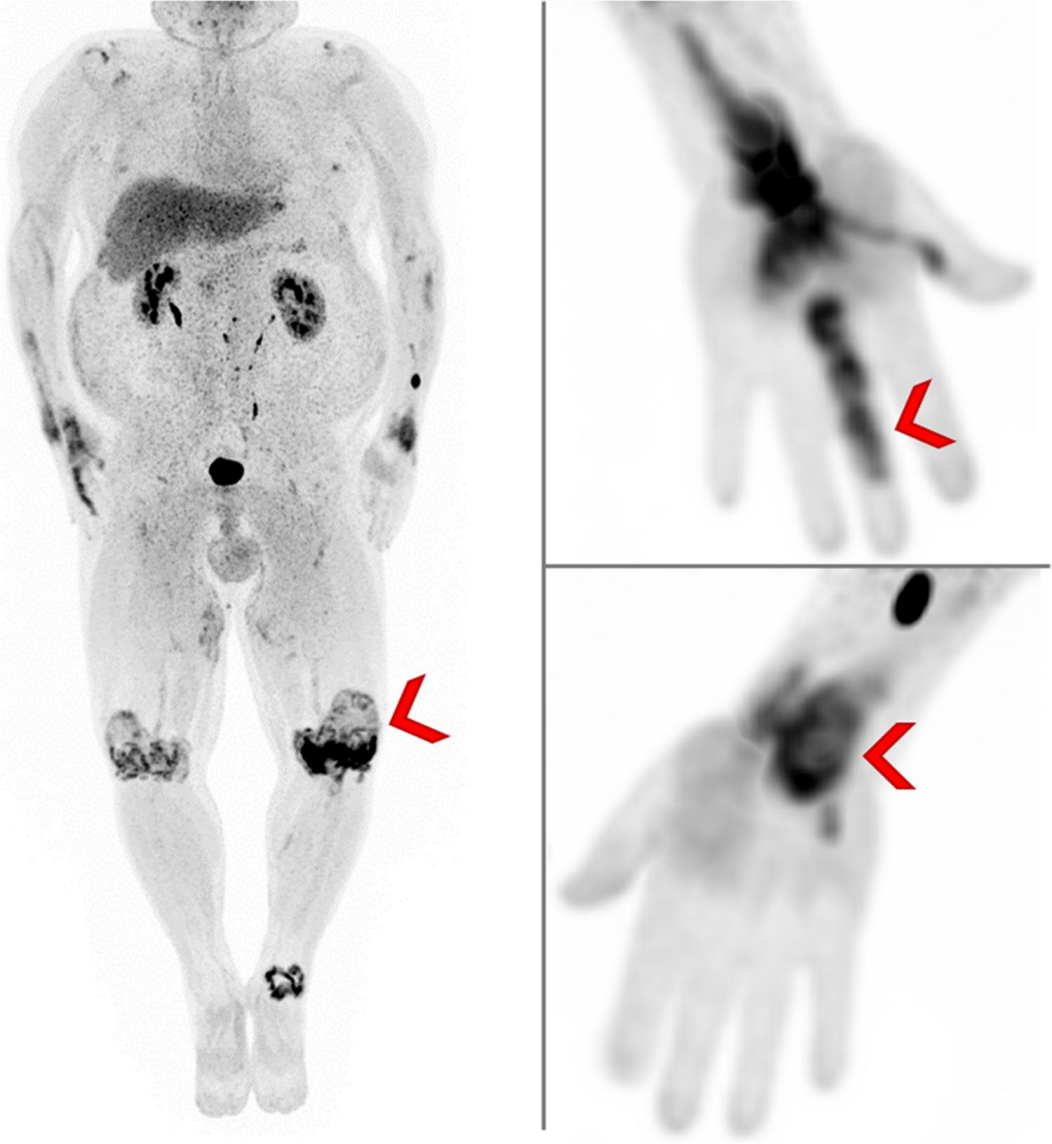
Maximum intensity projection of a 62-year-old patient suffering from rheumatoid arthritis that was examined with.^68^Ga-FAPI PET/CT. PET/CT imaging demonstrates tracer accumulation in multiple joints (marked with red arrows)

Rheumatoid arthritis (RA) is a chronic systemic autoimmune inflammatory disorder that primarily involves synovial joints. If not properly controlled, it leads to joint erosions and destruction. In RA, the treatment strategy is based on the control of synovitis and the prevention of joint injury. General principles of patient management include the early diagnosis of RA as well as the use of disease-modifying antirheumatic drugs and a treat-to-target strategy [28, 29]. For maintaining control of RA, it is essential to regularly assess disease activity, e.g., every 3 months. The clinical assessment of disease activity includes physical examination of the joints, the measurement of acute-phase reactant levels in the blood as well as patients’ reporting disease activity and quality of life [30]. Progression of disease is defined as tissue destruction occurring between two patient examinations, meaning that disease activity is measured only indirectly by progression of existing tissue damage. Direct measurement of disease activity is not established in clinical practice and may lack reproducibility [31]. Instead, PET/CT imaging using FAPIs may serve as a reliable, reproducible, and objective indicator of disease activity. FAPI PET/CT is capable of detecting activated fibroblast-like synoviocyte cells that have a central role in the pathogenesis of RA (Fig. 1). These cells, located in the lining and sublining of the synovium, contribute to pannus formation and the destruction of articular cartilage and bone.

## Search strategy

The search strategy involved a combination of relevant keywords and MeSH terms to retrieve all relevant studies on FAPI PET/CT for diagnosis of inflammatory arthritis. The electronic databases used in the search included PubMed, Scopus, ScienceDirect, and Google Scholar, and the search was restricted to articles published in the English language up to July 1, 2024, without any geographic or time restrictions. The search strategy included the following keywords: (“arthritis” OR “rheumatoid” OR “autoimmune”) AND (“PET/CT”) AND ((“FAPI”) OR (“fibroblast activation protein”)) AND (“imaging”). The search terms were combined using Boolean operators: “AND” and “OR” to ensure the inclusion of all relevant articles. There were no publications with non-significant/negative/null outcomes that were left out or excluded from this review.

To evaluate the potential role of FAPI PET/CT in the diagnosis of RA, Luo et al. performed a prospective study comparing the diagnostic accuracy of ^68^Ga FAPI- and ^18^F-FDG PET/CT in assessing joint disease activity [32]. A total of 20 patients with moderate to high disease activity of RA were recruited and underwent both ^68^Ga-FAPI- and ^18^F-FDG PET/CT within 1 week. Following imaging, all patients underwent treatment for RA. Clinical assessment of disease activity was based on a physical examination of the joints including tender joint count and swollen joint count. Furthermore, laboratory measures and patient self-reporting of physical function assessment were considered. The combined output of both PET/CT techniques detected 244 affected joints all of which demonstrated positive results at ^68^Ga-FAPI PET/CT. Fifteen of 244 (6.1%) FAPI-avid joints in six of 20 (30%) participants were not detected with ^18^F-FDG PET/CT. While the maximum standardized uptake value of the most affected joint in each participant was higher in ^68^Ga-FAPI than in ^18^F-FDG PET/CT, the maximum standardized uptake values of the joints at both ^68^Ga-FAPI and ^18^F-FDG PET/CT were both positively correlated with laboratory evaluation of C-reactive protein levels. Three participants underwent follow-up ^68^Ga-FAPI and ^18^F-FDG PET/CT after 6–10 months of treatment. According to the American College of Rheumatology/European League Against Rheumatism response criteria, two of these subjects with a good response and remission of the disease showed reduced uptake of ^68^Ga-FAPI and ^18^F-FDG in their joints [33]. The authors concluded that ^68^Ga-FAPI PET/CT might have a potential role for the assessment of RA since ^68^Ga-FAPI PET/CT demonstrated a higher number of affected joints compared to ^18^F-FDG PET/CT as well as a significant correlation with clinical and laboratory disease markers.

Remission rates for treatment in RA depend on a treatment regimen. For example, the remission rate of methotrexate ranges between 30 and 50%, while up to 40% of patients have unsatisfactory control of disease symptoms with disease-modifying antirheumatic drugs [34, 35]. Furthermore, the frequency of side effects and the treatment’s cost and accessibility must also be considered [36]. Thus, the identification of biomarkers that can predict treatment response prior to drug exposure currently has high priority. Pan et al. conducted a prospective cohort study regarding the use of ^68^Ga-FAPI PET/CT for the prediction of treatment response in RA [37]. A total of 19 patients underwent ^68^Ga-FAPI-04- and ^18^F-FDG PET/CT at baseline followed by tight control treatment against RA. Patients were followed up for treatment response at 3 months and 6 months after initiation of treatment with disease-modifying antirheumatic drugs. The core variables of disease activity included the clinical disease activity index and the simplified disease activity index. In addition, the number of PET-positive joints was recorded and the metabolic synovitis volume, as well as the total synovitis uptake. The total synovitis uptake, the metabolic synovitis volume, and the PET joint count in ^68^Ga-FAPI-04 and ^18^F-FDG PET/CT of the responders were significantly higher than those in non-responders. ^68^Ga-FAPI-04 PET/CT demonstrated a greater number of affected joints and higher tracer uptake in RA compared with ^18^F-FDG PET/CT. The baseline tracer uptake values in ^68^Ga-FAPI-04 PET/CT were significantly higher in early responders than in non-responders suggesting a potential role for the prediction of treatment response in RA for FAPI PET imaging.

Besides ^68^Ga-labeled compounds, FAPI tracers labeled by ^18^F are under investigation since ^18^F offers a higher image resolution owing to its lower end-point positron energy and longer half-life. Zhang et al. investigated the feasibility of ^18^F-FAPI-04 for the evaluation of arthritic progression and therapeutic response in experimental arthritis [38]. Fibroblast-like synoviocytes were obtained from patients with RA or osteoarthritis, and the relationship between ^18^F-FAPI-04 uptake and the inflammatory activity of RA fibroblast-like synoviocytes was investigated. Additionally, collagen-induced arthritis mice models were established and treated with methotrexate or etanercept. The results of PET imaging were compared by assessing macroscopic arthritic scores and histological staining. The severity of inflammation in affected joints correlated significantly with tracer uptake. Furthermore, an increase in ^18^F-FAPI-04 uptake in inflamed joints could be found before the deformity of the parental joints could be observed by histological examination. The sensitivity of ^18^F-FAPI-04 PET imaging in predicting progression or remission was significantly better than clinical arthritis scoring.

**Fig. 2:**
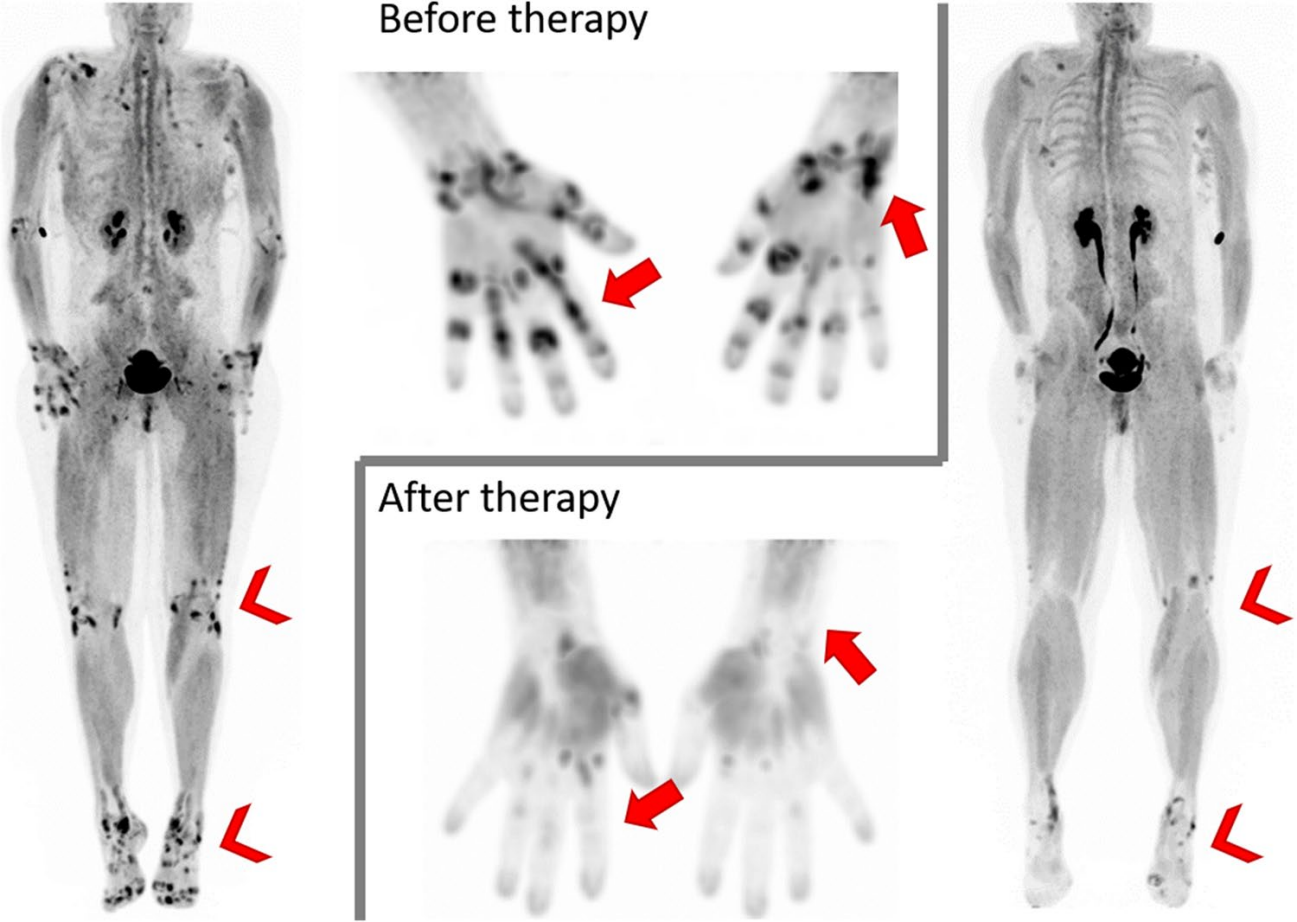
Maximum intensity projection of a 49-year-old patient that was examined with ^68^Ga-FAPI PET/CT suffering from rheumatoid arthritis before and after therapy with Blinatumomab (compassionate use), demonstrating excellent therapeutic response

Fibroblast activation is a central step in the extensive tissue response occurring in the various forms of arthritis. However, little is known about whether fibroblast activation is altered by the treatment of arthritis-resolving inflammation. Targeted drugs for arthritis, such as cytokine inhibitors, are designed to block the activation of adaptive and innate immune cells and do not directly address fibroblasts [39, 40]. Successful treatment of arthritis might also switch the phenotype of fibroblasts to a more homeostatic phenotype during the resolution phase. However, it is speculative whether such processes occur in patients and how such a switch from a pro-inflammatory to a pro-resolving fibroblast population might be controlled from a molecular perspective. To overcome this limitation, Rauber et al. sought to establish an in vivo imaging technique to detect fibroblast activation in the joints of humans and to test whether anti-cytokine treatment reduces inflammation in tissues [41]. Consequently, they investigated the link between fibroblast activation and a FAP signal determined by FAPI PET/CT to identify a molecular switch in fibroblasts occurring during the resolution of experimental arthritis and subsequently validated this molecular switch in fibroblasts during the resolution of human arthritis. They performed ^68^Ga-FAPI-04 PET/CT in 120 patients with inflammatory joint disease including rheumatoid arthritis (*n* = 20), psoriatic arthritis (*n* = 50), and axial spondyloarthritis (*n* = 50). Tracer accumulation was found in the joints and entheses of the upper and lower extremities and in the spine with similar maximum standardized uptake values. Mesenchymal cell activation mainly concurred with signs of inflammation as measured by simultaneous magnetic resonance imaging and correlated with validated composite scores of clinical disease activity in the respective diseases. To test whether the FAPI signal was associated with a fibroblast response related to structural damage, regions were further stratified based on the presence of erosions and/or osteoproliferative changes on MRI and CT as part of the PET scan. While FAPI tracer uptake was low to absent in inflammatory sites without structural changes, it was high in the lesions with erosions and osteoproliferation. Longitudinal FAPI-PET/CT scans in 34 patients who started treatment with either TNFi or interleukin (IL)−17A inhibitors (IL-17i) revealed a significant reduction and often even complete abolition of the FAP signal after cytokine blockade. In a sex-matched and age-matched comparative analysis, the reduction of the FAPI signal was significantly greater after IL-17i than with TNFi. These data provide evidence that mesenchymal activation is reversible during resolution of inflammation, suggesting that resident fibroblasts may change their functional pattern and may acquire a pro-resolving phenotype. To functionally test the role of key inflammatory cytokines on mesenchymal activation in the joints, FAPI-PET was performed in preclinical arthritis models and correlated with histological analysis for changes related to inflammation and damage. As in human arthritis, blockade of TNF and IL-17A significantly reduced FAP tracer uptake in the joints, again with more pronounced effects for IL-17i than for TNFi treatment. These data suggest that resolution of both human and experimental arthritis is associated with a change in mesenchymal activation during resolution of inflammation. To address the nature of this fibroblast phenotype alteration, mRNA expression in all fibroblast subtypes using single-cell mRNA sequencing (scRNA-seq) was analyzed. The data thus obtained suggest a remodeling of the mesenchymal compartment during the resolution of inflammation in humans. This process is associated with the emergence of a pro-resolving network built around a CD200-expressing fibroblast phenotype that is also present in human arthritis during the resolution phase. Consistent with previous reports, the data show that administration of CD200-Fc, which mimics the effects of pro-resolving fibroblasts, controls inflammation and tissue damage in experimental arthritis [42]. Thus, CD200-Fc may provide a new therapeutic option to efficiently promote a pro-resolving environment in the joint and allow to restore tissue homeostasis in arthritis. This is of utmost importance since to date therapies that directly target fibroblasts are not available for the treatment of chronic inflammatory diseases [43, 44]. The authors concluded that PET scans using FAPI tracers are a suitable method to monitor the changes in the mesenchymal network during inflammation in humans in vivo and allow to disentangling of destructive from pro-resolving fibroblasts (Fig. 2).

**Fig. 3:**
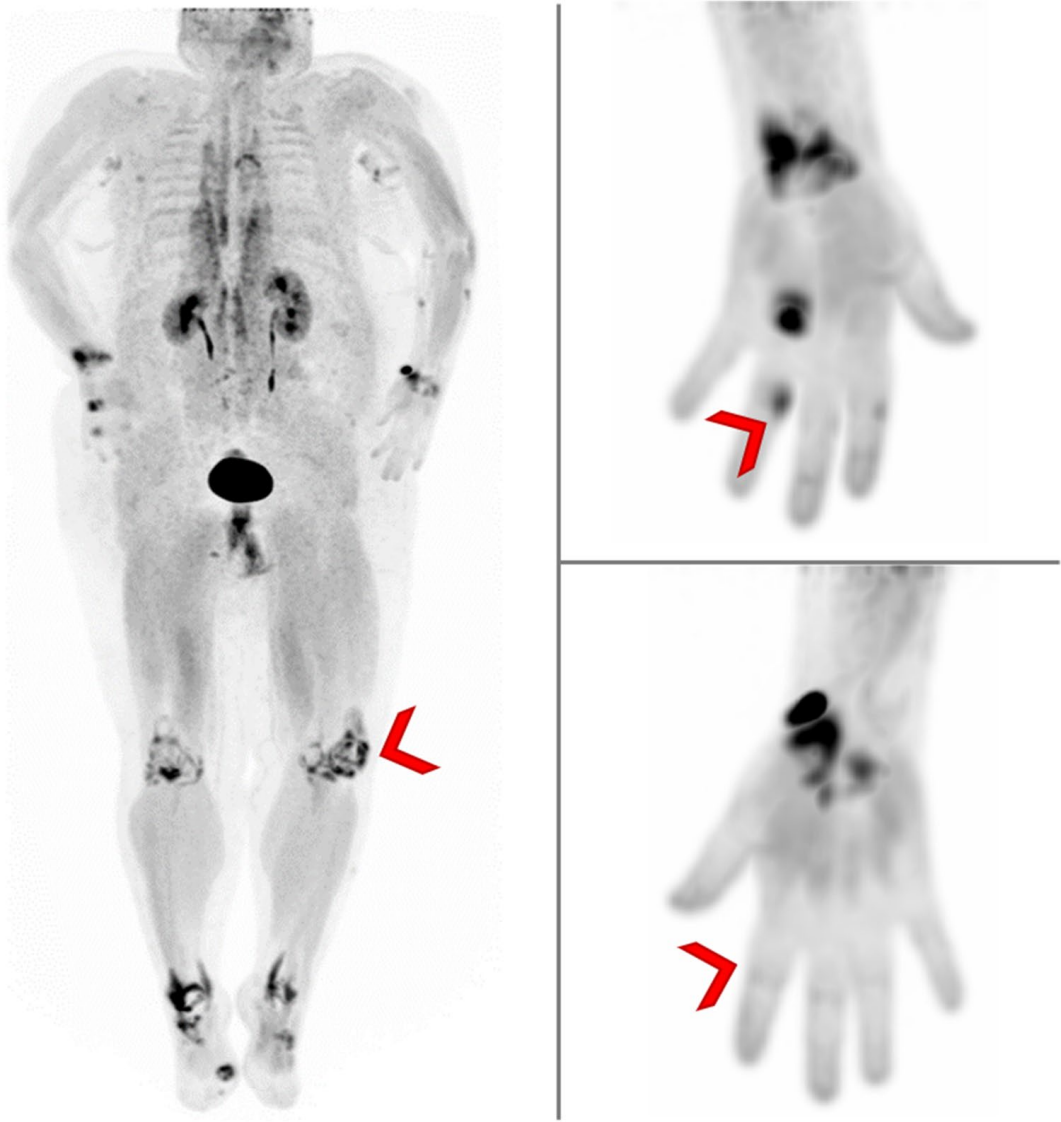
Maximum intensity projection of a 50-year-old patient suffering from psoriasis arthritis that was examined with.^68^Ga-FAPI PET/CT. Imaging demonstrates tracer accumulation in multiple joints (marked with red arrows)

Besides RA, one of the most common inflammatory joint diseases is psoriatic arthritis (PsA), affecting up to 30% of patients with psoriasis [45]. To identify patients with psoriasis that have a high risk of transition to PsA is crucial as these require a closer follow-up and could benefit from preventive measures. The majority of patients with psoriasis who develop PsA undergo a prodromal state characterized by increasing non-specific musculoskeletal complaints and beginning functional limitations, in which inflammation remains subclinical [46, 47]. In the early stage of PsA, patients usually present with unspecific arthralgia, arthralgia fatigue, and stiffness, as well as inflammatory changes on imaging, but lack evidence of clinical arthritis [48, 49]. Several studies in patients with psoriasis before the onset of PsA demonstrated evidence of synovitis, tenosynovitis, enthesitis, and enthesophytes on MRI scans, ultrasound, or CT scans that were associated with a high risk of transition from psoriasis to PsA [50–52]. To date, the molecular mechanisms of this transition are not fully understood and conventional imaging modalities as well as soluble biomarkers do not provide sufficient insight into the early molecular alterations that predate inflammation and structural damage [53]. To evaluate whether there is evidence for early fibroblast activation in the joints and entheses of patients with psoriasis that have a high risk of developing PsA, Corte et al. conducted a prospective cohort study using ^68^Ga-FAPI-04 PET/CT [54]. These authors investigated the hypothesis that the distribution of tracer uptake correlates with clinical and ultrasound findings and that the presence of articular tracer uptake in patients with psoriasis is associated with the development of PsA. A total of 36 patients with a biopsy-proven or dermatologist-confirmed diagnosis of psoriasis and arthralgia were prospectively recruited. All patients were examined clinically and by musculoskeletal ultrasound to rule out past or present clinical or imaging signs of inflammatory joint involvement related to PsA. Furthermore, patients with psoriasis were not allowed to fulfill the Classification Criteria for Psoriatic Arthritis, and rheumatoid factor and anti-citrullinated protein antibodies had to be negative in all participants [55]. ^68^Ga-FAPI-04 uptake was found in 318 (7.9%) joints and 369 (7.3%) entheses in 29 (80.6%) participants. A significant positive relationship was found between ^68^Ga-FAPI-04-PET/CT signal intensity on the one hand and the tender joint count and the tender entheses count on the other, while there was no correlation with ultrasound findings. Kaplan–Meier analyses were conducted in a subset of 17 patients for whom a follow-up of at least 6 months after the ^68^Ga-FAPI-04-PET/CT scan was available. Of the 13 patients with increased ^68^Ga-FAPI-04 tracer accumulation, only 2 were not diagnosed with PsA during the 38-week observation period. In total, 11 of these 13 patients had received a diagnosis of PsA with the presence of both clinical and imaging (i.e., ultrasound, MRI) findings compatible with PsA during this period. Thus, patients with relevant synovial and entheseal ^68^Ga-FAPI-04 uptake had a significantly higher risk of developing PsA. Subgroup analysis with respect to the presence versus absence of ultrasound changes did not reveal significant differences indicating that a positive ^68^Ga-FAPI-04-PET/CT signal predicts progression to PsA independently of ultrasound findings. These results suggest that the earliest signs in the joints and entheses of patients with psoriasis transitioning to PsA are hallmarked by the activation of tissue-resident mesenchymal cells and can be detected by ^68^Ga-FAPI-04-PET/CT. The observation that patients with psoriasis both with and without ultrasound-detected inflammation progress to PsA if a positive ^68^Ga-FAPI-04-PET/CT signal is present shows that mesenchymal activation may precede inflammation in the course to PsA. This underlines the potential of using mesenchymal cell targeting in the prevention and therapy of early psoriatic disease (Fig. 3).

Periprosthetic joint infection (PJI) is one of the most devastating complications of joint arthroplasty. Successful treatment of PJI is based on prompt and accurate diagnosis of this condition. Nuclear medicine examinations including bone scintigraphy and white blood cell scanning are frequently used for the evaluation or exclusion of PJI [56]. Furthermore, several studies explored the application of ^18^F-FDG PET/CT under the rationale that the activation of inflammatory cells such as leucocytes or macrophages is accompanied by an increase in glucose utilization, leading to hypermetabolism of inflammatory foci [57, 58]. In an exploratory study, Wang et al. assessed the efficiency of ^68^Ga-FAPI-04 PET/CT in diagnosing PJI [59]. A total of 103 patients that were hospitalized due to symptomatic hip arthroplasty were enrolled in this retrospective single-center study. Twenty-eight of these subjects were diagnosed with PJI. Two diagnostic criteria, SUVmax and uptake pattern, were used to diagnose PJI and compared to serological tests. The application of radiomics was also attempted. The sensitivity and specificity of SUVmax were 100 and 72%. The area under the curve was 0.898 which was better than that of all serological tests. The sensitivity, specificity, and accuracy of the uptake pattern were 100, 93.1, and 95%, respectively. The radiomics analysis yielded significantly different imaging features compared to aseptic failure. The results of this pilot study are promising; however, one of the limitations of this investigation is that no comparison was made with established nuclear medicine methods such as bone scan, white blood cell scanning, or ^18^F-FDG PET/CT.

## Limitations

This paper has numerous limitations. To date, the literature on the use of FAPI PET/CT in inflammatory arthritis is still limited. The studies are often pilot studies with small numbers of patients without longer follow-up periods. So far, there are no randomized double-blind studies investigating the use of FAPI PET/CT, e.g., for therapy response. Some studies were conducted retrospectively and without comparison with a gold standard.

## Conclusion

The development of FAPIs suitable for PET/CT has been a breakthrough in molecular imaging. Besides their successful use in oncologic disease, the studies currently available in inflammatory joint diseases suggest that the direct targeting of activated fibroblasts afforded by this innovative technology has great diagnostic potential. Diagnosis of mesenchymal activation before irreversible joint damage and early non-invasive monitoring of targeted drug therapies seem possible with this new imaging approach and are expected to improve patient care in the future. However, pertinent evidence is still scarce, relying predominantly on the study of small patient cohorts or retrospective analyses. Prospective randomized controlled trials are needed to better define the clinical role of FAPI PET imaging in rheumatic disease and to fully exploit the considerable potential of this novel imaging technique also in this field.
